# Effect of Proton Pump Inhibitor Therapy on NOX5, mPGES1 and iNOS expression in Barrett’s Esophagus

**DOI:** 10.1038/s41598-019-52800-7

**Published:** 2019-11-07

**Authors:** Dan Li, Deepthi Deconda, Aihua Li, Fadlallah Habr, Weibiao Cao

**Affiliations:** 1 Department of Medicine, Rhode Island Hospital and Warren Alpert Medical School of Brown University, Providence, RI 02903 USA; 2 Department of Pathology, Rhode Island Hospital and Warren Alpert Medical School of Brown University, Providence, RI 02903 USA; 3 Southcoast Physicians Group, Fall river, MA 02720 USA

**Keywords:** Cancer prevention, Barrett oesophagus, Translational research

## Abstract

Acid reflux may contribute to the progression from Barrett’s esophagus (BE) to esophageal adenocarcinoma (EA). However, it is not clear whether the molecular changes present in BE patients are reversible after proton pump inhibitor (PPI) treatment. In this study we examined whether PPI treatment affects NOX5, microsomal prostaglandin E synthase (mPGES)-1 and inducible nitric oxide synthase (iNOS) expression. We found that NADPH oxidase 5 (NOX5), mPGES-1 and iNOS were significantly increased in BE mucosa. One-month PPI treatment significantly decreased NOX5, mPGES1 and iNOS. In BAR-T cells, NOX5 mRNA and p16 promoter methylation increased after pulsed acid treatment in a time-dependent manner. Four or eight-week-acid induced increase in NOX5 mRNA, NOX5 protein and p16 methylation may be reversible. Twelve-week acid treatment also significantly increased NOX5, mPGES1 and iNOS mRNA expression. However, twelve-week-acid-induced changes only partially restored or did not recover at all after the cells were cultured at pH 7.2 for 8 weeks. We conclude that NOX5, mPGES1 and iNOS may be reversible after PPI treatment. Short-term acid-induced increase in NOX5 expression and p16 methylation might be reversible, whereas long-term acid-induced changes only partially recovered 8 weeks after removal of acid treatment.

## Introduction

How the progression from Barrett’s esophagus (BE) to esophageal adenocarcinoma (EA) occurs is not fully understood. We have reported that NOX5-S is identifiable in Barrett’s cells BAR-T and EA cells FLO-1 and OE33^1,2^ and is significantly increased in FLO-1 cells and EA tissues^3^. NOX5-S mRNA is also elevated in Barrett’s mucosa with high-grade dysplasia^4^. NOX5-S expression and H_2_O_2_ production are significantly enhanced in response to pulsed acid treatment in BAR-T and OE33 cells^1^ and Barrett’s mucosa^4^. These data suggest that NOX5-S may be responsible for the overproduction of reactive oxygen species (ROS) in BE and in EA cells. How NOX5-S is upregulated in EA cells is not known. Multiple factors may contribute to it, e.g. acid, bile acid, acid/bile acid reflux-induced inflammation, and others. We have reported that Rho kinase, ERK1/2 MAP kinases and cAMP response element binding protein are involved in acid-induced increase in NOX5-S expression^4,5^. Platelet activating factor, which may be produced after acid exposure, may activate signal transducer and activator of transcription 5 (STAT5) and then upregulate NOX5-S^6^. NOX5-S may play an important role in acid-induced increase in cell proliferation in Barrett’s cells BAR-T and EA cells (OE33 and FLO-1)^1,7^. NOX5-S-mediated increase in cell proliferation may be dependent on the activation of COX2^8^ and microsomal prostaglandin E synthase 1 (mPGES1)^7^, and on the reduction of p16 via promoter methylation^1^. NOX5-S may also be involved in the acid-induced DNA damage^9^. These data suggest that persistent acid reflux present in BE patients may significantly increase NOX5-S expression, ROS production, cell proliferation and DNA damage, thereby contributing to the progression from BE to dysplasia and to EA. These data suggest that reactive oxygen species may be important in this progression. However, whether NOX5 is reversible upon PPI treatment is not known.

COX-2-derived prostaglandin E_2_ may also contribute to the progression from BE to EA since (1) PGE_2_ increases cell proliferation, promoter methylation and tumor growth^10^; (2) selective COX-2 inhibitors may prevent EA development in a rat model of BE^11^. PGE_2_ is produced by PGE synthase (PGES), which has three isoforms: a cytosolic (cPGES) and two microsomal (mPGES) isomerases^12–14^. Microsomal PGES1 has been reported to be increased in an animal model of BE^15^ and in human EA^16^. We have also reported that mPGES1 is identifiable in FLO-1 EA cells and that mPGES1 mRNA and protein levels are significantly enhanced in response to pulsed acid treatment in FLO-1 cells^7^. Therefore, mPGES1 may also contribute to the development of EA. It is not known whether PPI treatment may reverse mPGES1.

It is known that nitric oxide (NO) is involved in angiogenesis, apoptosis, gene expression, and DNA damage and may be important in carcinogenesis and tumor progression in the gastrointestinal tract^17,18^. NO may produce peroxynitrite and N_2_O_3_ when it reacts with the superoxide radical and oxygen, respectively^19^. Three isoforms of NO synthase have been identified: endothelial NO synthase (NOS), neuronal NOS and inducible NOS. Large amount of NO may be generated by inducible NOS during inflammation and may in part mediate the progression from BE to EA^20,21^.

Our aim in this study is to examine NOX5, mPGES1 and iNOS in BE and to investigate whether PPI treatment reverses these genes. We found that NOX5, mPGES1 and iNOS were significantly increased in BE mucosa. PPI treatment for one month significantly decreased all three genes.

## Results

### NADPH oxidases in BE mucosa

Eight male BE patients aged from 58 to 75 (average 67.5 ± 2.3) were enrolled in this study. The duration of the disease was 3–17 years (11.2 ± 2.1 years). The length of the BE was 2–11 cm (4.6 ± 1 cm). BE patients were asked to discontinue PPI for one month and then the first biopsy was obtained. After the first biopsy, the treatment with proton pump inhibitor (PPI) was started twice a day for one month. At the end of this one-month period of PPI treatment, biopsies were repeated.

**Figure 1:**
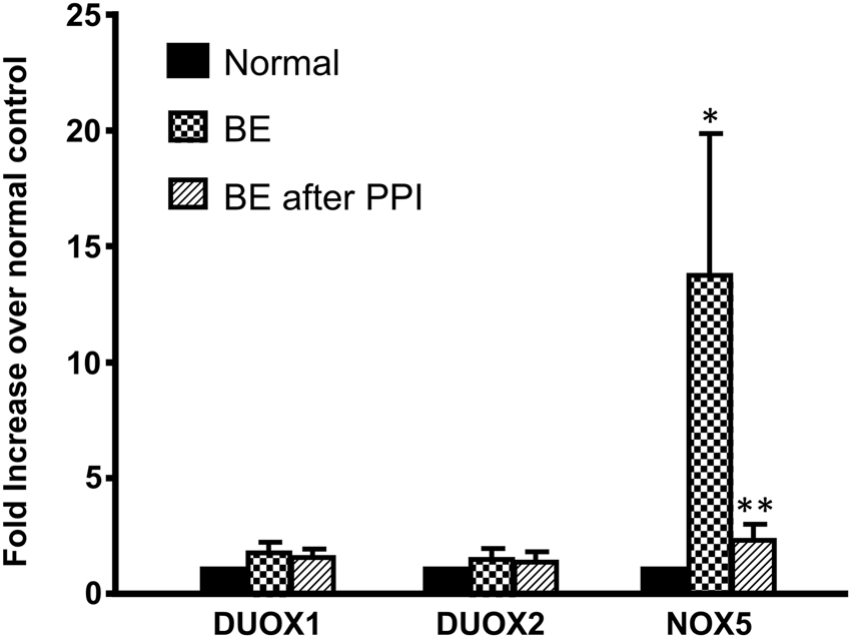
Effect of PPI treatment on the expression of NADPH oxidases. NOX5 mRNA was significantly increased, when compared with normal esophageal mucosa. DUOX1 and DUOX2 mRNAs did not have significant changes between BE mucosa and normal esophageal mucosa. PPI treatment for a month significantly decreased NOX5 mRNA. The data suggest that the increased NOX5 mRNA may be reversible in BE mucosa after PPI treatment. N = 5; ANOVA *P < 0.05, compared normal, **P < 0.05, compared with BE without PPI

**Figure 2:**
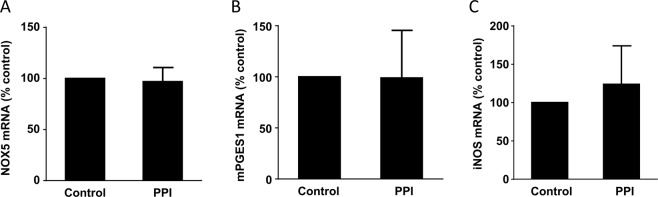
Expression of NOX5, mPGES1 and iNOS in normal esophageal mucosa (**A**). PPI treatment did not have any effect on NOX5 in normal esophageal mucosa. (**B**) PPI treatment did not have any effect on mPGES1 in normal esophageal mucosa. (**C**) PPI treatment did not have any effect on iNOS in normal esophageal mucosa.

Figure 1 showed that NOX5 mRNA was significantly increased in BE mucosa, when compared with normal esophageal mucosa. Dual oxidase 1 (DUOX1) and DUOX2 mRNAs did not have significant changes between BE mucosa and normal esophageal mucosa. PPI treatment for a month significantly decreased NOX5 mRNA in BE mucosa. However, PPI did not have any effect on NOX5 mRNA in normal esophageal mucosa (Fig. 2A). The data suggest that the increased NOX5 mRNA may be reversible in BE mucosa after PPI treatment.

### Microsomal PGE synthase 1 in BE mucosa

**Figure 3:**
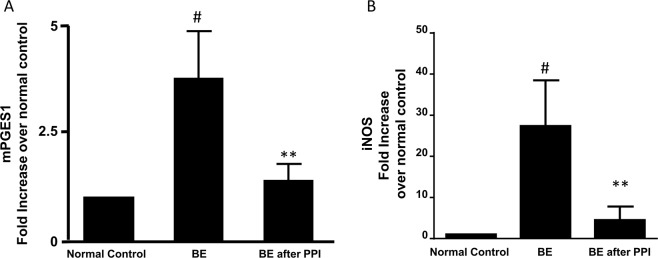
Expression of mPGES1 and iNOS. (**A**) mPGES1 mRNA was significantly increased in BE mucosa, an increase which was significantly decreased by PPI treatment, suggesting that mPGES1 may be reversible in BE mucosa by PPI treatment. (**B**) Inducible NOS was significantly increased in the BE mucosa, an increase which was significantly reduced by PPI treatment, suggesting that the overexpression of iNOS might be reversible. N = 5; ANOVA ^#^P < 0.02, compared normal, **P < 0.05, compared with BE without PPI.

We have previously reported that mPGES1 is identifiable in FLO-1 EA cells and is increased in response to pulsed acid treatment^7^. Figure 3A showed that mPGES1 mRNA was significantly increased in BE mucosa, an increase which was significantly attenuated by PPI treatment, suggesting that mPGES1 may be reversible in BE mucosa after PPI treatment. PPI did not have any effect on mPGES1 in normal esophageal mucosa (Fig. 2B).

### Inducible nitric oxide synthase in BE mucosa

Inducible NOS expression has been shown to be gradually upregulated in the progression from BE to EA, but not in normal esophageal/gastric mucosa^20,21^. We found that inducible NOS was significantly increased in the BE mucosa, an increase which was significantly reduced by PPI treatment (Fig. 3B), suggesting that the overexpression of iNOS might be reversible in BE mucosa after PPI treatment. PPI did not have any effect on iNOS in normal esophageal mucosa (Fig. 2C).

### Effect of omeprazole on NOX5, mPGES1 and iNOS expression in Barrett’s EA cells

**Figure 4:**
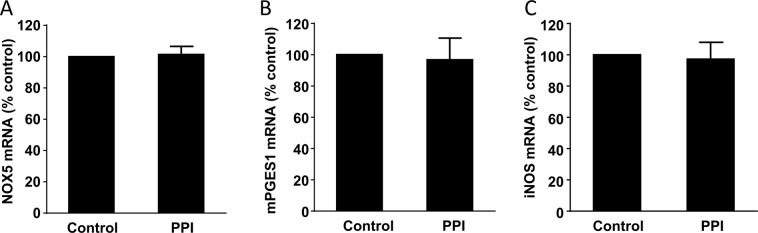
Effect of omeprazole on NOX5, mPGES1 and iNOS in FLO-1 cells. FLO-1 cells were treated with omeprazole 1 μM for 24 hours and then mRNAs were measured by real-time PCR. A. Omeprazole had no effect on NOX5 mRNA. (**B**) Omeprazole had no effect on mPGES1 mRNA. (**C**) Omeprazole had no effect on iNOS mRNA.

Barrett’s EA cell line FLO-1 was treated with omeprazole 1μM for 24 hours and then mRNAs were measured by real-time PCR. Figure 4 showed that omeprazole had no effect on NOX5, mPGES1 and iNOS mRNA expression.

### Effect of long-term acid treatment on NOX5-S expression, p16 promoter methylation, mPGES1 and iNOS expression

We have previously shown that NADPH oxidase NOX5-S exists in FLO-1 EA cells and that it is upregulated in Barrett’s mucosa with high-grade dysplasia and responsible for the acid-induced ROS production^4^.

**Figure 5:**
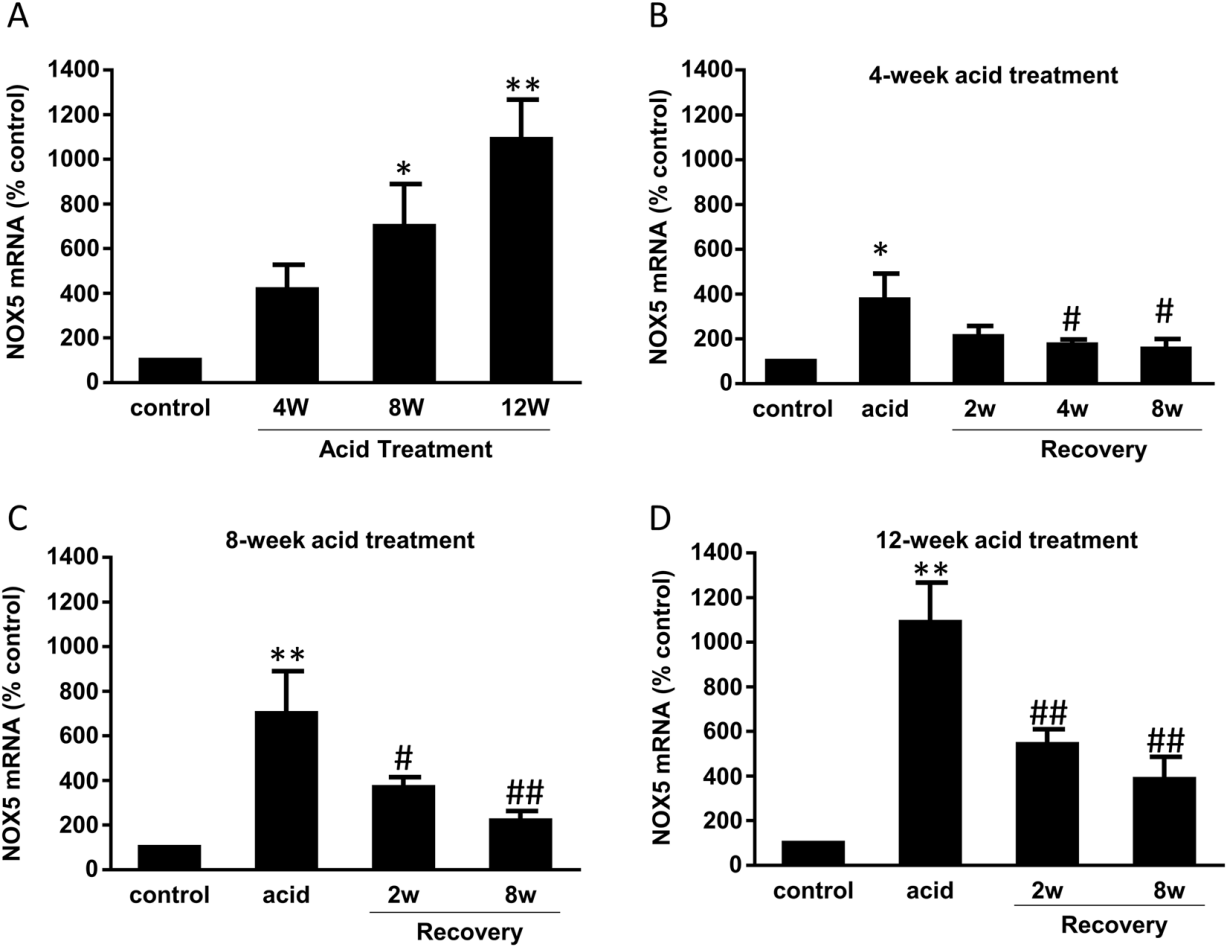
Effect of long-term acid treatment on NOX5-S mRNA expression. BAR-T cells were first treated with acidic culture medium (pH 4.0) for 5 min, three times a day for 2, 4, 8 and 12 weeks. Then BAR-T cells were cultured at normal culture medium (pH 7.2) for additional 2, 4 and 8 weeks after acid treatment for 2, 4, 8 and 12 weeks, respectively. (**A**) NOX5 mRNA increased after pulsed acid treatment in a time-dependent manner. (**B**) The increase in NOX5 mRNA induced by pulsed acid treatment for 4 weeks almost completely recovered after the cells were cultured in normal culture medium for 4–8 weeks. (**C**) The increase in NOX5 mRNA induced by pulsed acid treatment for 8 weeks almost completely recovered after the cells were cultured in normal culture medium for 8 weeks. (**D**) 12-week-acid-induced increase in NOX5 mRNA only partially restored after cells were cultured in normal culture medium for 8 weeks. These data suggest that, if BAR-T cells exposed to pulsed acid treatment for a longer period of time, the cells would take a much longer time to recover. N = 3, ANOVA. *P < 0.01, **P < 0.001, compared with control, ^#^P < 0.05, ^##^P < 0.01 compared with acid group.

**Figure 6:**
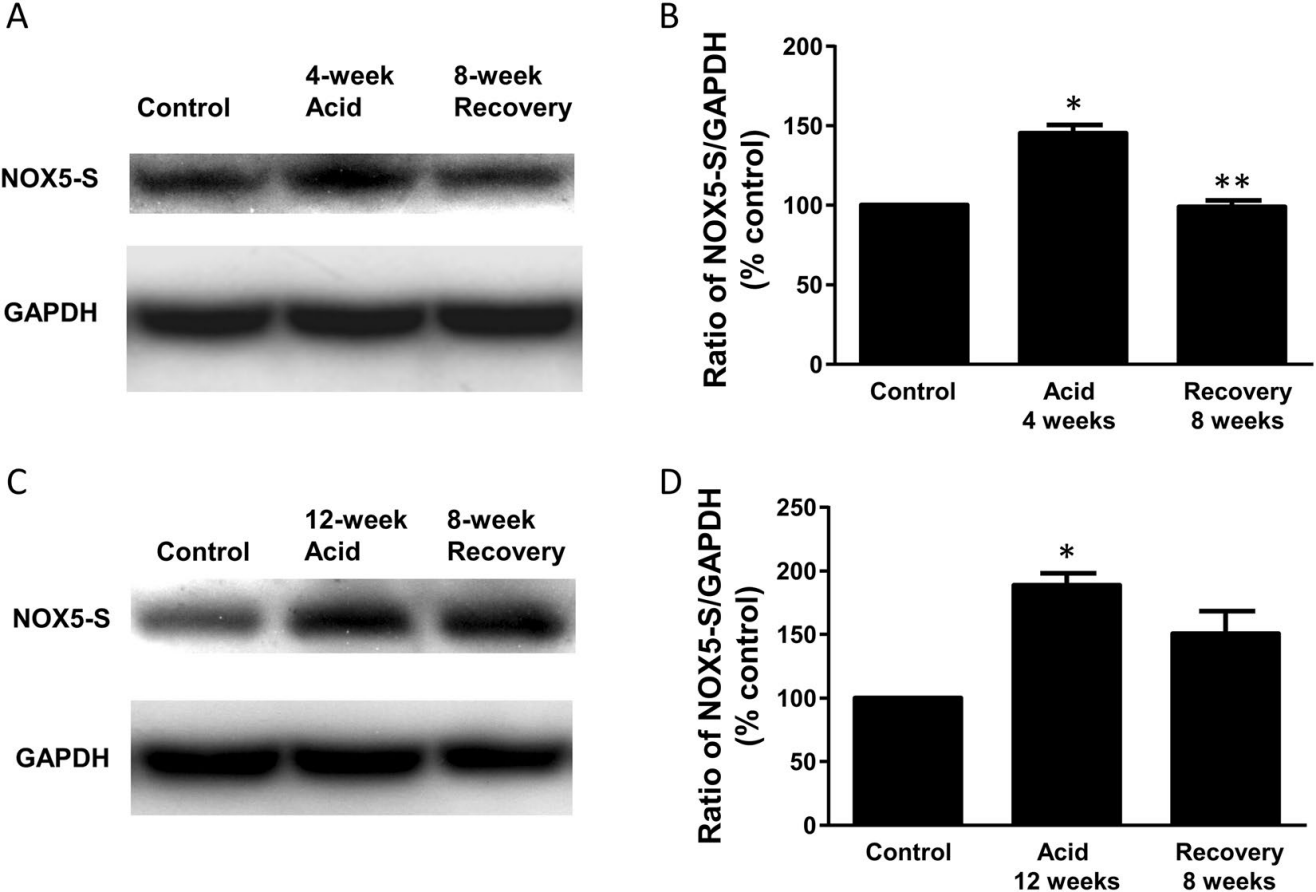
Effect of long-term acid treatment on NOX5-S protein expression. BAR-T cells were first treated with acidic culture medium (pH 4.0) for 5 min, three times a day for 4 and 12 weeks. Then BAR-T cells were cultured at normal culture medium (pH 7.2) for additional 8 weeks. (**A**) Typical image of three Western blot analysis and (**B**) Summarized data showed that the increase in NOX5 protein induced by pulsed acid treatment for 4 weeks almost completely recovered after the cells were cultured in normal culture medium for 8 weeks. (**C**) Typical image of three Western blot analysis and (**D**) summarized data showed that the increase in NOX5 protein induced by pulsed acid treatment for 12 weeks only partially restored after the cells were cultured in normal culture medium for 8 weeks. These data suggest that, if BAR-T cells exposed to pulsed acid treatment for a longer period, the cells would take a much longer time to recover. N = 3, ANOVA. *P < 0.01, compared with control, **P < 0.01 compared with acid group. Original Western blot images are available in the supplemental file at 10.1038/s41598-019-52800-7.

To examine whether the overexpression of NOX5-S is reversible, BAR-T cells were first treated with acidic culture medium (pH 4.0) for 5 min, three times a day for 2, 4, 8 and 12 weeks. Then BAR-T cells were cultured at normal culture medium (pH 7.2) for additional 2, 4 and 8 weeks after acid treatment for 2, 4, 8 and 12 weeks, respectively. Figure 5A showed that NOX5 mRNA increased after pulsed acid treatment in a time-dependent manner. Figure 5B and 5c showed that the increase in NOX5 mRNA induced by pulsed acid treatment for 4 and 8 weeks almost recovered after cells were cultured in normal culture medium for 8 weeks. However, 12-week-acid-induced increase in NOX5 mRNA only partially restored after the cells were cultured in normal culture medium for 8 weeks (Fig. 5D). Similarly, pulsed acid treatment for 4 and 12 weeks significantly increased NOX5-S protein levels. The increase in NOX5-S protein induced by pulsed acid treatment for 4 weeks almost recovered after the cells were cultured in normal culture medium for 8 weeks. However, 12-week-acid-induced increase in NOX5 protein only partially restored after the cells were cultured in normal culture medium for 8 weeks (Fig. 6). These data suggest that, if BAR-T cells exposed to pulsed acid treatment for a longer period, the cells would take a much longer time to recover.

**Figure 7:**
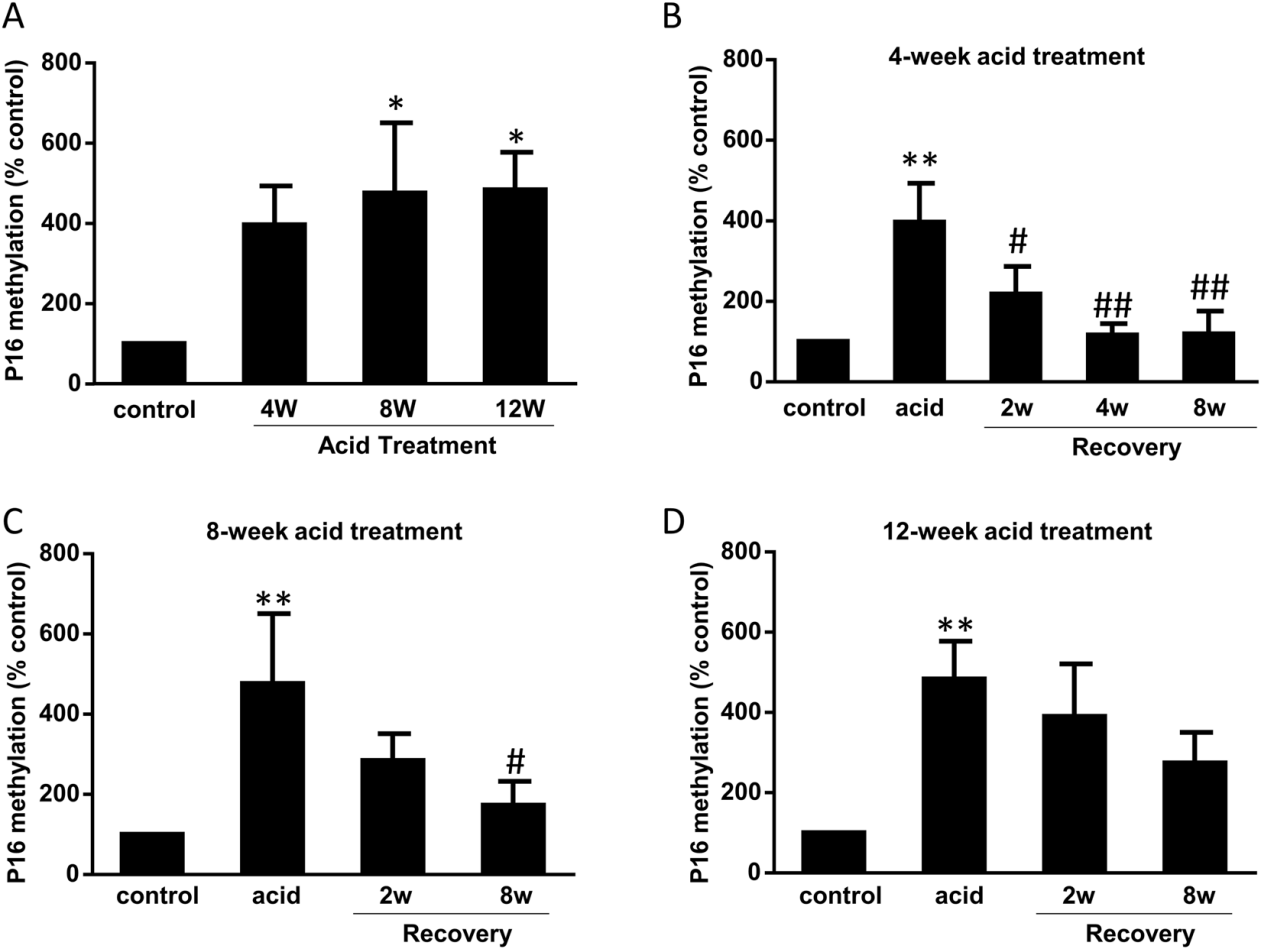
Effect of long-term acid treatment on p16 methylation (**A**). p16 promoter methylation increased after pulsed acid treatment in a time-dependent manner. (**B**) This increase in p16 promoter methylation induced by pulsed acid treatment for 4 weeks almost completely recovered after cells were cultured in normal culture medium for 4–8 weeks. (**C**) The increase in p16 promoter methylation induced by pulsed acid treatment for 8 weeks almost completely recovered after the cells were cultured in normal culture medium for 8 weeks. (**D**) 12-week-acid induced increase in p16 promoter methylation did not restore after the cells were cultured in normal culture medium for 8 weeks. N = 3, ANOVA, *P < 0.05, **P < 0.01, compared with control; ^#^P < 0.05, ^##^P < 0.01, compared with acid group.

**Figure 8:**
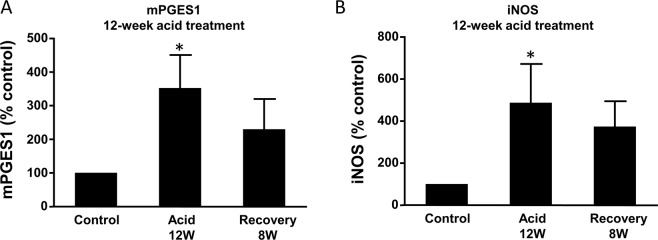
Effect of long-term acid treatment on mPGES1 and iNOS mRNA expression. BAR-T cells were first treated with acidic culture medium (pH 4.0) for 5 min, three times a day for 12 weeks. Then BAR-T cells were cultured at normal culture medium (pH 7.2) for additional 8 weeks after acid treatment for 12 weeks. (**A**) 12-week acid treatment significantly increased mPGES1 mRNA. The increase did not restore after the cells were cultured in normal culture medium for 8 weeks. (**B**) 12-week acid treatment significantly increased iNOS mRNA. The increase did not restore after the cells were cultured in normal culture medium for 8 weeks. ANOVA. *P < 0.05, compared with control.

Similarly, p16 promoter methylation increased after pulsed acid treatment in a time-dependent manner (Fig. 7A). This increase in p16 promoter methylation induced by pulsed acid treatment for 4 and 8 weeks almost recovered after the cells were cultured in normal culture medium for 8 weeks (Fig. 7B,C). However, 12-week-acid-induced increase in p16 promoter methylation did not restore after the cells were cultured in normal culture medium for 8 weeks (Fig. 7D). In addition, twelve-week acid treatment significantly increased mPGES1 and iNOS mRNA expressions (Fig. 8). These increases did not restore after the cells were cultured in normal culture medium for 8 weeks. These data suggest that long-term acid treatment may cause irreversible gene changes.

## Discussion

We have previously reported that NOX5-S is responsible for the increase in cell proliferation in response to acid treatment in Barrett’s cells BAR-T and EA cells (OE33 and FLO-1)^1,7^, and is involved in acid-induced DNA damage^9^. In this study we examined whether PPI reverses oxidative stress genes.

We found that NOX5 and iNOS were markedly elevated in BE mucosa. We have previously reported that NOX5-S is the major isoform of NADPH oxidases in FLO-1 EA cells. The increase of NOX5 and iNOS may increase the production of ROS and thereby cause gene damage. Reactive oxygen species may increase gene mutation and modify the functions of enzyme and proteins (e.g. activation of oncogene products and/or inhibition of tumor suppressor proteins) via the damage of DNA, RNA, lipids and proteins^22,23^. Microsomal PGES1 mRNA was also significantly increased in BE mucosa. Similarly, pulsed acid treatment significantly enhanced the expressions of NOX5, iNOS, and mPGES1 mRNAs in BAR-T cells.

We also found that NOX5, mPGES1 and iNOS may be reversible in BE mucosa after PPI treatment since PPI treatment significantly downregulated the NOX5 mRNA, mPGES1 mRNA and iNOS mRNA expression. However, PPI did not affect these gene expression in normal esophageal mucosa.

To further confirm these *in vivo* data, we examined whether the overexpression of NOX5-S and hypermethylation of p16 may be reversible *in vitro*. We found that short-term (such as 4 weeks) acid-induced increase in NOX5 mRNA and p16 promoter methylation may be reversible since the increase in NOX5 mRNA and p16 methylation induced by pulsed acid treatment for 4 and 8 weeks almost completely recovered after the cells were cultured in normal culture medium for 8 weeks. However, long-term (such as 12 weeks) acid treatment-induced increase in NOX5 mRNA, iNOS, mPGES1, and p16 methylation may take more than 8 weeks to recover since these genes were not restored after the cells were cultured in normal culture medium for 8 weeks.

PPI has both anti-acid secretion and anti-inflammatory effects^24^. Our *in vitro* data suggest that inhibition of acid reflux by PPI may be involved in the reversal of NOX5, mPGES1 and iNOS *in vivo* since 1) short-term (such as 4 weeks) acid-induced increase in NOX5 mRNA and p16 promoter methylation was recovered after removal of acid treatment; 2) Omeprazole did not have any effect on the expression of NOX5, mPGES1 and iNOS in cultured FLO-1 cells.

In conclusion, NOX5, mPGES1 and iNOS were significantly increased in BE mucosa. Proton pump inhibitor treatment for one month significantly decreased these three gene expression. In BAR-T cells, NOX5 mRNA, iNOS mRNA, mPGES1 mRNA and p16 promoter methylation increased after pulsed acid treatment. Four or eight-week-acid induced increase in NOX5 mRNA and p16 methylation may be reversible. However, twelve-week acid-induced increase in NOX5 mRNA, iNOS mRNA, mPGES1 mRNA and p16 methylation only partially recovered 8 weeks after removal of acid treatment. Our data imply that early PPI treatment might be important in the prevention of the irreversible molecular changes induced by acid reflux.

## Methods

### PPI treatment and esophageal biopsies

Eight male BE patients aged from 58 to 75 (average 67.5 ± 2.3) were enrolled in this study. The duration of the disease was 3–17 years (11.2 ± 2.1 years). The length of the BE was 2–11 cm (4.6 ± 1 cm). First biopsy was obtained after the patients discontinued PPI for one month. Four specimens every 2 centimeters were obtained within the length of the Barrett’s esophagus. Biopsies from the normal-looking squamous mucosa of the upper esophagus (approximately 10 cm above BE) were used as control. Then the treatment with proton pump inhibitor (PPI) omeprazole was started 40 mg, twice a day for one month. At the end of this one-month period of PPI treatment, biopsies were repeated. The experimental protocols were approved by the Human Research Institutional Review Committee at Rhode Island Hospital and at Providence VA hospital. All research was performed in accordance with relevant guidelines and informed consent was obtained from all participants.

### Cell culture

Human Barrett’s cell line BAR-T^25^, provided to us by Dr. Rhonda Souza (University of Texas Southwestern Medical Center, Texas), was cultured in Keratinocyte Medium-2 (Ca^2+^-free solution, Cambrex, Rockland, ME) supplemented with 1.8 mM CaCl_2_ and other agents as we previously reported^1^. For acid treatment, BAR-T cells were treated with acidic culture medium (pH 4.0) for 5 min, three times a day for 2, 4, 8 and 12 weeks. Cells were collected after acid treatment. To examine whether the molecular changes are reversible, cells were cultured at normal culture medium (pH 7.2) for additional 2, 4 and 8 weeks after acid treatment for 2, 4, 8 and 12 weeks, respectively.

BE EA cell line FLO-1 was generously provided to us by Dr. David Beer (University of Michigan). FLO cells were cultured in DMEM containing 10% fetal bovine serum and antibiotics. For PPI treatment, FLO-1 cells were treated with omeprazole 1μM for 24 hours.

### Reverse transcription-PCR

Total RNA from esophageal biopsies was purified by using The RNeasy Micro Kit (Qiagen, Germantown, MD). TRIzol reagent (ThermoFisher Scientific, Foster City, CA) was used to purify total RNA from the cultured cells and GeneAmp Gold RNA PCR reagent kit (ThermoFisher Scientific, Foster City, CA) was utilized to reversely transcribe 1.5 *μ*g of total RNAs.

### PCR array

96-well PCR array was performed by using Human Oxidative Stress PCR Array (Qiagen) according to the manufacturer’s protocol. Data were analyzed by using Qiagen web-based software. All the data were normalized by five house-keeping genes.

### Quantitative real-time PCR

Real time PCR was performed as we previously described^2,5,26^. The primers used were: NOX5-S sense (5′-AAGACTCCATCACGGGGCTGCA-3′), NOX5-S antisense (5′-CCTTCAGCACCTTGGCCAGA-3′); iNOS sense (5′-AGTGACACAGGATGACCTTCAG-3′)^27^, iNOS antisense (5′-GGGTTGCATCCAGCTTGACCA-3′); mPGES1 sense (5′-GGGGTCTTGGGTTCCTGTAT-3′), mPGES1 antisense (5′-GACTGCAGCAAAGACATCCA-3′); 18S sense (5′-CGGACAGGATTGACAGATTGATAGC-3′) and 18S antisense (5′-TGCCAGAGTCTCGTTCGTTATCG-3′). Reactions were carried out in an Applied Biosystems StepOnePlus™ Real-Time PCR System for 40 cycles at 95 °C for 45 s, 58 °C for 50 s, and 72 °C for 50 s.

### Bisulfite conversion of DNA sample

A CpG modification Kit (EZ DNA methylation-Direct^TM^ Kit, Zymo Research, CA) was used to convert genomic DNA according to the manufacturer’s protocol and as we previously described^1^.

### Conventional methylation specific PCR (MSP)

MSP was performed as we previously described^1^. The primers for the bisulfite-converted methylated sequence were *p16*MF:5′-TTATTAGAGGGTGGGGCGGATCGC-3′ and *p16*MR:5′-GACCCCGAACCG CGACCGTAA-3′. The primers for the bisulfite-converted unmethylated sequence were *p16*UF:5′-TTATTAGAGGGTGGGGTGGATTGT-3′ and *p16*UR:5′-CAACCCCAAACCACAACCATAA-3′.

Real-Time Quantitative Methylation Specific PCR was performed as we previously described^1^. Methylated *p16* gene was detected by using primers *p16*MF and *p16*MR. β-actin served as a control. Primers for β-actin were: β-actin-F: 5′-TGGTGATGGAGGAGGTTTAGTAAGT-3′ and β-actin-R: 5′-AACCAATAAAACCTACTCCTCCCTTAA-3′. 40 cycles of PCR were run at 95 °C 30 s, 60 °C for 30 s and 72 °C for 30 s.

#### Western blot analysis

Western blot analysis was performed as described previously^28^. The NOX5 antibody was generously provided to us by Dr. David Lambeth^29^ and used at a dilution of 1:1000. The same membrane was used to probe GAPDH. The GAPDH antibody was used at a dilution of 1:1000.

### Statistical analysis

Data were expressed as mean ± S.E. Analysis of variance (ANOVA) was used to test statistical differences among multiple groups and Fisher’s protected least significant difference test was utilized to check significance.

## Supplementary information

Supplemental information

## Supplementary information

is available this paper at 10.1038/s41598-019-52800-7.
