# Supplementary material for: Effect of Proton Pump Inhibitor Therapy on NOX5, mPGES1 and iNOS expression in Barrett’s Esophagus
Source: Sci Rep. 2019 Nov 7;9:16242. doi: 10.1038/s41598-019-52800-7 (PMC6838155; doi:10.1038/s41598-019-52800-7)
Supplement: Supplementary file 1 — Supplemental information [file 41598_2019_52800_MOESM1_ESM.pdf]

Effect of Proton Pump Inhibitor Therapy on NOX5, mPGES1 and iNOS expression in Barrett's Esophagus

Dan Li[*, 1], Deepthi Deconda[*, 3], Aihua Li[1], Fadlallah Habr[†, 1], Weibiao Cao[†, 1, 2]

[1]Department of Medicine, Rhode Island Hospital and Warren Alpert Medical School of Brown University, Providence, RI 02903, USA (DL, WC, FH); [2]Department of Pathology, Rhode Island Hospital and Warren Alpert Medical School of Brown University, Providence, RI 02903, USA (WC); [3]Southcoast Physicians Group, Fall river, MA 02720 (DD).

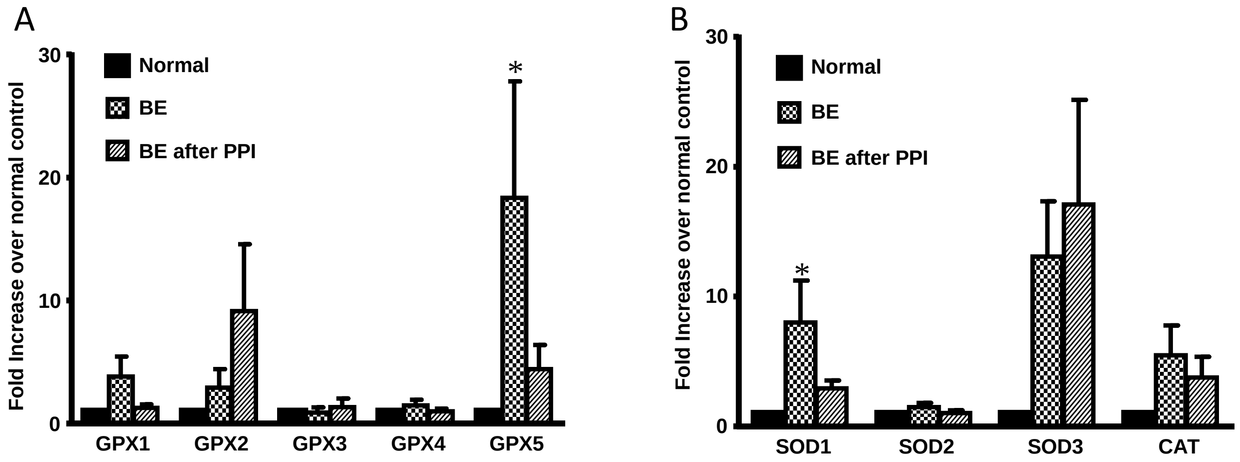

Expression of glutathione Peroxidases (GPX), superoxide dismutase (SOD) and catalase (CAT) in BE mucosa A. GPX5 mRNA was significantly increased in BE mucosa, when compared with normal esophageal mucosa. Although PPI treatment decreased GPX5, the change did not reach statistical significance. GPX1, GPX2, GPX3 and GPX4 did not have any significant difference between BE and normal esophageal mucosa and between with and without PPI treatment. B. SOD1 mRNA was significantly increased in BE mucosa. The decrease of SOD1 mRNA induced by PPI treatment did not reach statistical significance. SOD2, SOD3 and catalase did not have statistically significant differences between BE and

normal esophageal mucosa and between with and without PPI treatment. N=5, ANOVA * P<0.05, compared normal.

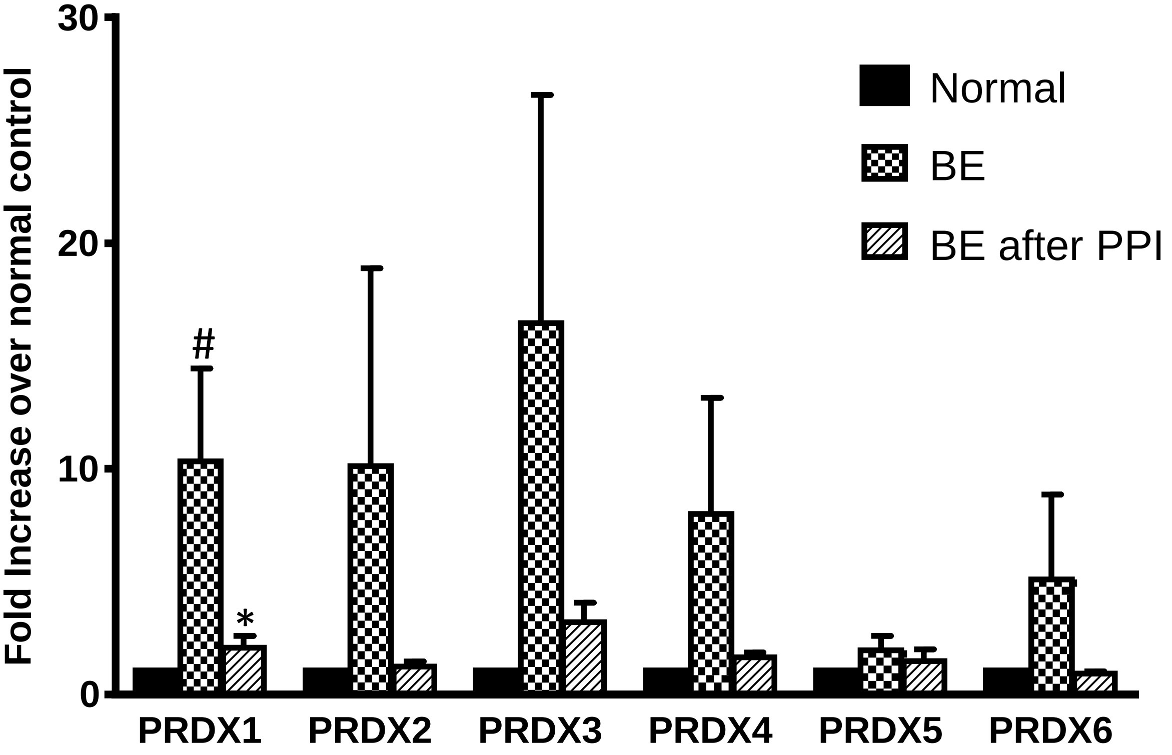

Effect of PPI treatment on peroxiredoxins (PRDXs) PRDX1 mRNA was significantly increased in BE mucosa, an increase which was significantly decreased by PPI treatment. PRDX2, PRDX3, PRDX4, PRDX5 and PRDX6 were also increased in BE mucosa, but the change did not have statistical significance. N=5; ANOVA # P<0.05, compared normal, * P<0.05, compared with BE without PPI.

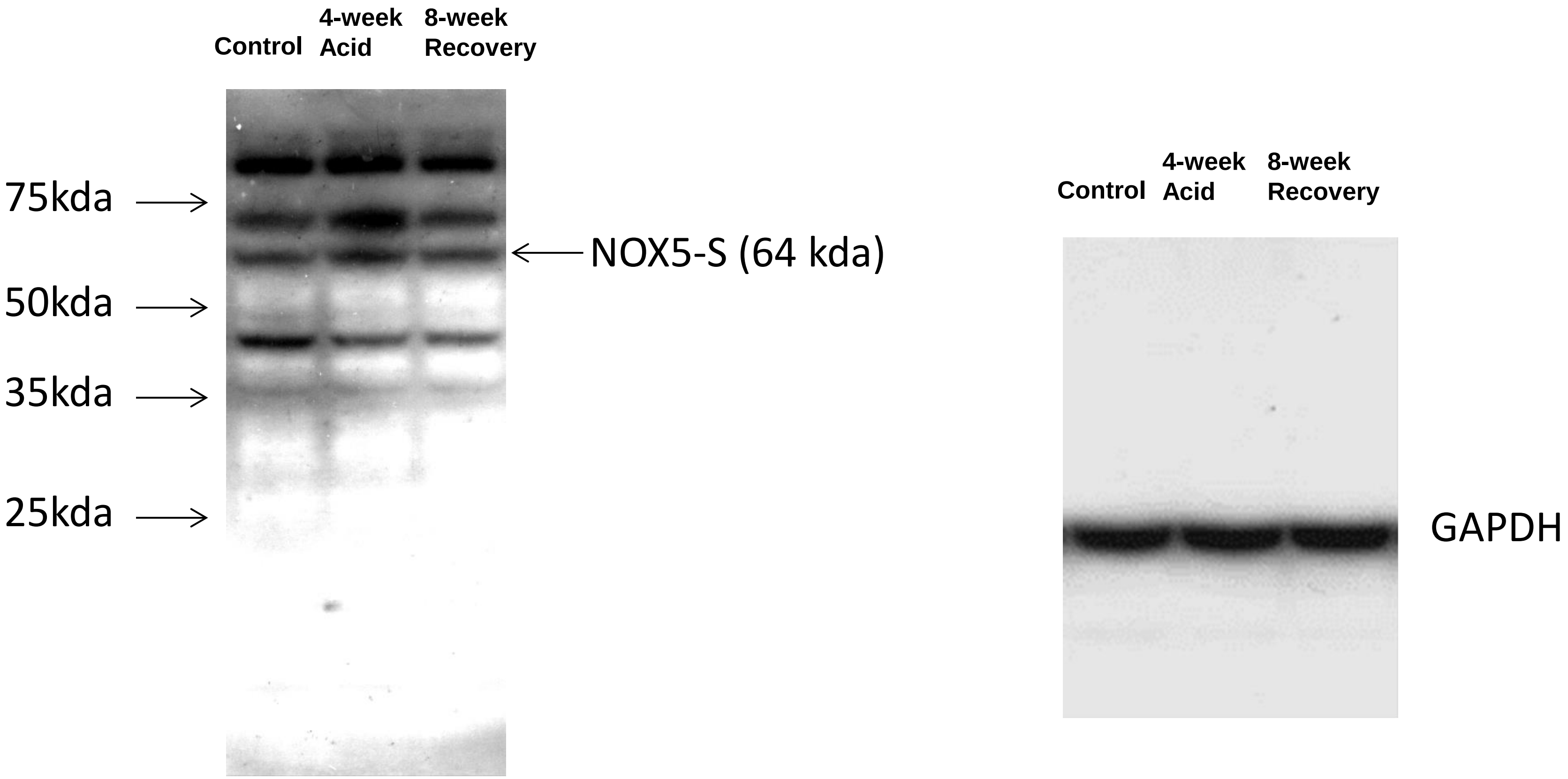

Full gel for figure 6A

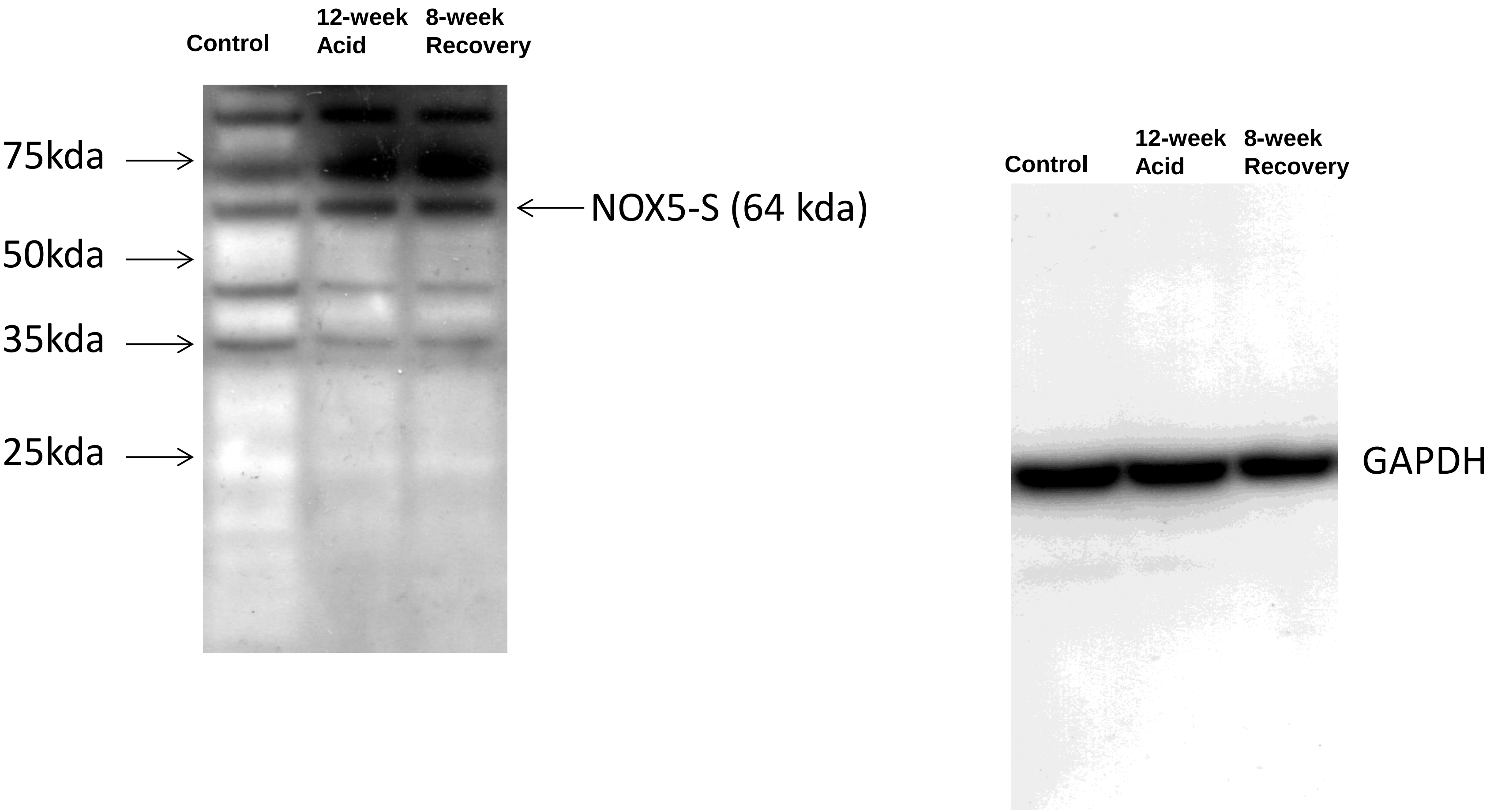

Full gel for figure 6B
